# Supplementary material for: Empirical methods for controlling false positives and estimating confidence in ChIP-Seq peaks
Source: BMC Bioinformatics. 2008 Dec 5;9:523. doi: 10.1186/1471-2105-9-523 (PMC2628906; doi:10.1186/1471-2105-9-523)
Supplement: Additional File 1 — A variety of html documents from the USeq web site detailing the available applications, their best usage, output file type descriptions, command line menus, etc. [file 1471-2105-9-523-S1.zip › USeqUserGuides/JavaDoc.zip!/JavaDoc/meme/package-use.html]

Uses of Package meme


|  |  |  |
| --- | --- | --- |
| **Overview** \| **Package** \| Class \| **Use** \| **Tree** \| **Deprecated** \| **Index** \| **Help** | |  |
| PREV   NEXT | **FRAMES**    **NO FRAMES**     **All Classes** |  |


---


## **Uses of Package meme**

| Packages that use meme | |
| --- | --- |
| **meme** | Wrapper for meme tweaked for processing multiple rounds of SELEX oligos. |

Classes in meme used by meme

****MemeMotif****
  
          This contains info generated from parsing a meme report, each found motif is represented by this class.

****MemeParser****
  
          Parses meme output into MemeMotif objects and stores some generalize info re the meme run.

****MemeResults****
  
          Holds the results from various parts of MemeR.

****MotifHit****
  
          Holds data related to a particular match of a matrix to a sequence.

---


|  |  |  |
| --- | --- | --- |
| **Overview** \| **Package** \| Class \| **Use** \| **Tree** \| **Deprecated** \| **Index** \| **Help** | |  |
| PREV   NEXT | **FRAMES**    **NO FRAMES**     **All Classes** |  |


---
